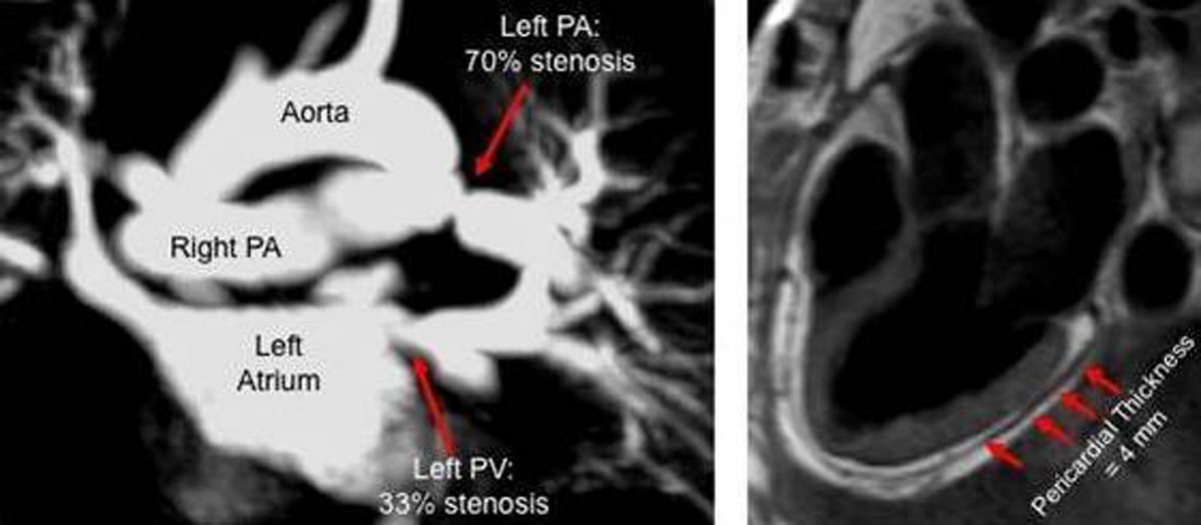# Utility of CMR in identification of post lung transplant cardiovascular complications

**DOI:** 10.1186/1532-429X-14-S1-P84

**Published:** 2012-02-01

**Authors:** Farshad Forouzandeh, Kamil Muhyieddeen, Harish Seethamraju, Dipan J Shah

**Affiliations:** 1 The Methodist DeBakey Heart & Vascular Center and Baylor College of Medicine, Houston, TX, USA

## Background

Graft dysfunction after lung transplantation is typically attributable to infection or rejection. In the absence of these entities, structural cardiovascular (CV) abnormalities need to be sought. At our center this evaluation typically includes a myriad of diagnostic tests: echocardiography, chest computed tomographic angiography, nuclear medicine lung perfusion imaging, and right heart catheterization (RHC). This study was designed to evaluate the utility of a novel comprehensive CMR protocol for identification of all post-operative structural CV complications in lung transplant recipients.

## Methods

We enrolled 80 consecutive lung transplant recipients who were referred for CMR evaluation of CV abnormalities. There were 47 men and 33 women; mean age 56.7±13.6 years; 55 Caucasians, 9 Hispanic, 13 African American, and 3 Asian. Underlying restrictive disease was present in 39 subjects, obstructive disorders in 22, and other disorders in 19. Forty-three subjects had undergone double lung transplant, while 37 subjects had single lung transplant (23 left lung, and 14 right lung). The comprehensive CMR protocol included complete short and long axis cine imaging (SSFP) to evaluate cardiac size and function; ECG gated spin echo imaging to assess pericardial thickness; contrast enhanced MRA of the pulmonary arterial (PA) and venous (PV) systems to assess for anastomotic site stenosis; phase contrast velocity flow mapping to assess anastomotic site peak velocity and right and left PA flow fraction; and inversion recovery SSFP to evaluate for myocardial fibrosis.

## Results

All 80 subjects completed CMR imaging without difficulty and none were excluded from this analysis for reasons of image quality. The mean LVEF was 66.4±9.23%, mean RVEF was 54.37±10.45%, right and left PAs flow were 2.6±1.21 and 2.6± 1.31 liter/min, respectively. Specific complications identified and the prevalence is shown in Table 1. Patients with moderate or severe PA or PV anastomotic site stenosis underwent RHC and intravascular ultrasound, which confirmed CMR findings; patients with severe stenosis underwent stenting of the respective vessel. Patients with CMR evidence of pericardial constriction underwent pericardiectomy with improvement in symptoms.

**Table 1:** 

Complication	n (%)
PA Anastomotic Site Stenosis Moderate Stenosis (50-70%) Severe Stenosis (> 70%)	6 (8%) 2 (3%)
PV Anastomotic Site Stenosis Moderate Stenosis (50-70%) Severe Stenosis (> 70%)	1 (1%) 1 (1%)
Pericardial constriction	6 (8%)
Acquired pericardial cyst causing hemodynamic compromise	2 (3%)
Myocardial fibrosis	24 (30%)

## Conclusions

A comprehensive CMR protocol can be a useful single noninvasive test for the detection of structural cardiovascular (CV) abnormalities after lung transplantation.

## Funding

Not applicable.

**Figure 1:**